# Polymerized human hemoglobin increases the effectiveness of cisplatin-based chemotherapy in non-small cell lung cancer

**DOI:** 10.18632/oncotarget.27776

**Published:** 2020-10-20

**Authors:** Alfredo Lucas, Donald A. Belcher, Carlos Munoz, Alexander T. Williams, Andre F. Palmer, Pedro Cabrales

**Affiliations:** ^1^Department of Bioengineering, University of California San Diego, La Jolla, CA 92093, USA; ^2^William G. Lowrie Department of Chemical and Biomolecular Engineering, The Ohio State University, Columbus, OH 43210, USA

**Keywords:** chemotherapy, cisplatin, polymerized hemoglobin, non-small cell lung cancer, hypoxia

## Abstract

Cisplatin is a promising therapeutic for the treatment of non-small cell lung cancer (NSCLC). Unfortunately, a significant portion of NSCLC patients relapse due to cisplatin chemoresistance. This chemoresistance is thought to be primarily associated with hypoxia in the tumor microenvironment. Administration of hemoglobin (Hb)-based oxygen (O_2_) carriers (HBOCs) is a promising strategy to alleviate hypoxia in the tumor, which may make cisplatin more effective. In this study, we administered a high O_2_ affinity, relaxed state (R-state) polymerized hemoglobin (PolyHb) to three different NSCLC cell lines cultured *in vitro* and implanted *in vivo* into healthy mice. The R-state PolyHb administered in this study is unable to deliver O_2_ unless under severe hypoxia which significantly limits its oxygenation potential. *In vitro* sensitivity studies indicate that the administration of PolyHb increases the effectiveness of cisplatin under hypoxic conditions. Additional animal studies revealed that co-administration of PolyHb with cisplatin attenuated tumor growth without alleviating hypoxia. Analysis of reactive O_2_ species production in the presence of hypoxic culture indicates that exogenous ROS production by oxidized PolyHb may the mechanism of chemosensitization. This ROS mechanism, coupled with oxygenation, may be a potential chemosensitizing strategy for use in NSCLC treatment.

## INTRODUCTION

Non-small cell lung cancer (NSCLC) remains one of the leading causes of cancer death and constitutes ~80 to 85% of all types of lung cancers [1]. Approximately 40% of all newly diagnosed NSCLCs are in stage IV, for which cytotoxic combination chemotherapy is the first line of defense [2]. In many combination chemotherapy regimens for NSCLC, cisplatin is used in combination with etoposide [3]. Through large multicenter clinical trials, cisplatin-based combination therapy results in a moderate survival advantage for treating metastatic NSCLC [4, 5]. However, a significant fraction of patient relapses occur due to cisplatin chemoresistance. Therefore, it is necessary to find novel therapeutic approaches to improve the effects of cisplatin in treating NSCLC.

Cisplatin chemoresistance is associated with the development of hypoxia in the tumor microenvironment. Hypoxia-induced cisplatin resistance is studied extensively in NSCLC, and multiple mechanisms for its development have been suggested [6–9]. Many mechanisms suggested in the literature involve the expression of hypoxia-inducible factor 1α (HIF-1α). The expression of HIF-1α, in the presence of moderate hypoxia, activates a series of intracellular regulatory pathways that promote cell survival. Upon activation of HIF-1α, cells predominantly shift towards glycolytic synthesis of ATP, which is promoted by the overexpression of GLUT transporters. HIF-1α also promotes the increased synthesis of vascular endothelial growth factors (VEGF). These factors promote angiogenesis within the tumor microenvironment, which leads to increased tumor perfusion [10]. Endogenous reactive oxygen species (ROS) are also associated with the expression and stabilization of HIF-1α [11, 12].

Furthermore, the expression of HIF-1α is associated with the reduction of ROS in cancer cells, which promotes cancer cell survival [13]. Cisplatin’s mechanism of action involves DNA cross-linking and damage, which results in the initiation of cellular apoptotic pathways [14]. Other studies suggest that cisplatin’s cytotoxicity involves the formation of mitochondrial ROS, which can lead to the initiation of apoptotic pathways through mitochondrial membrane damage, as well as more widespread cellular damage [15–19]. Therefore, activation of protective mechanisms via expression of HIF-1α is likely to affect the efficacy of cisplatin in the presence of hypoxia.

In an attempt to reverse (i.e., oxygenate) the tumor’s hypoxic microenvironment, several studies have supplemented cisplatin treatment with hemoglobin-based oxygen carriers (HBOCs) [20–25]. HBOCs often involve chemically cross-linked acellular hemoglobin (Hb) and are capable of transporting oxygen (O_2_). Alterations in the biophysical properties of HBOCs designed to reduce hypoxia may lead to targeted O_2_ release in the tumor [26]. This targeted O_2_ release may alleviate hypoxia-induced cisplatin resistance. However, this is difficult to achieve in practice. The above studies have been capable of effectively increasing the sensitivity of cancerous cells to cisplatin, both *in vitro* and *in vivo*, via supplementation of HBOCs, and they attribute this gained efficacy to increased O_2_ delivery. However, the concentrations of HBOC used in previous studies were insufficient to provide a significant increase in tumor O_2_ delivery. This indicates that HBOC mediated tumor oxygenation is not the sole reason for the increased sensitivity to chemotherapy.

An alternative explanation for increased chemosensitivity in the presence of HBOCs is the accumulation of ROS in the tumor microenvironment as a result of Hb oxidation reactions. This latter hypothesis has not been directly evaluated in the existing cancer literature. Hence, this study explores both mechanisms, i. e., the increase in oxygenation of the tumor and the catalyzed formation of ROS by the HBOC, in *in vitro* and *in vivo* studies using low molecular weight (MW) polymerized human Hb (PolyHb) in the relaxed (R) quaternary state. R-state PolyHb has an extremely high O_2_ affinity (P_50_ ~1.5 mm Hg), which limits O_2_ release to only regions suffering from severe hypoxia. Thus, the mechanism of action of R-state PolyHb may be due to its role in ROS generation.

In this study, PolyHb was administered in conjunction with cisplatin to three different NSCLC cell lines cultured *in vitro* and implanted *in vivo* into healthy mice. HIF-1α and ROS expression in the presence and absence of PolyHb, without cisplatin, was assessed in hypoxic cell culture. The *in vitro* sensitivity to cisplatin, in the absence and presence of PolyHb at different concentrations, was also assessed through markers of apoptosis and necrosis, as well as through sensitivity analysis. Differences in the *in vivo* tumor volume were assessed longitudinally in the presence of cisplatin treatment with and without PolyHb supplementation.

## RESULTS

### PolyHb properties

**Figure 1:**
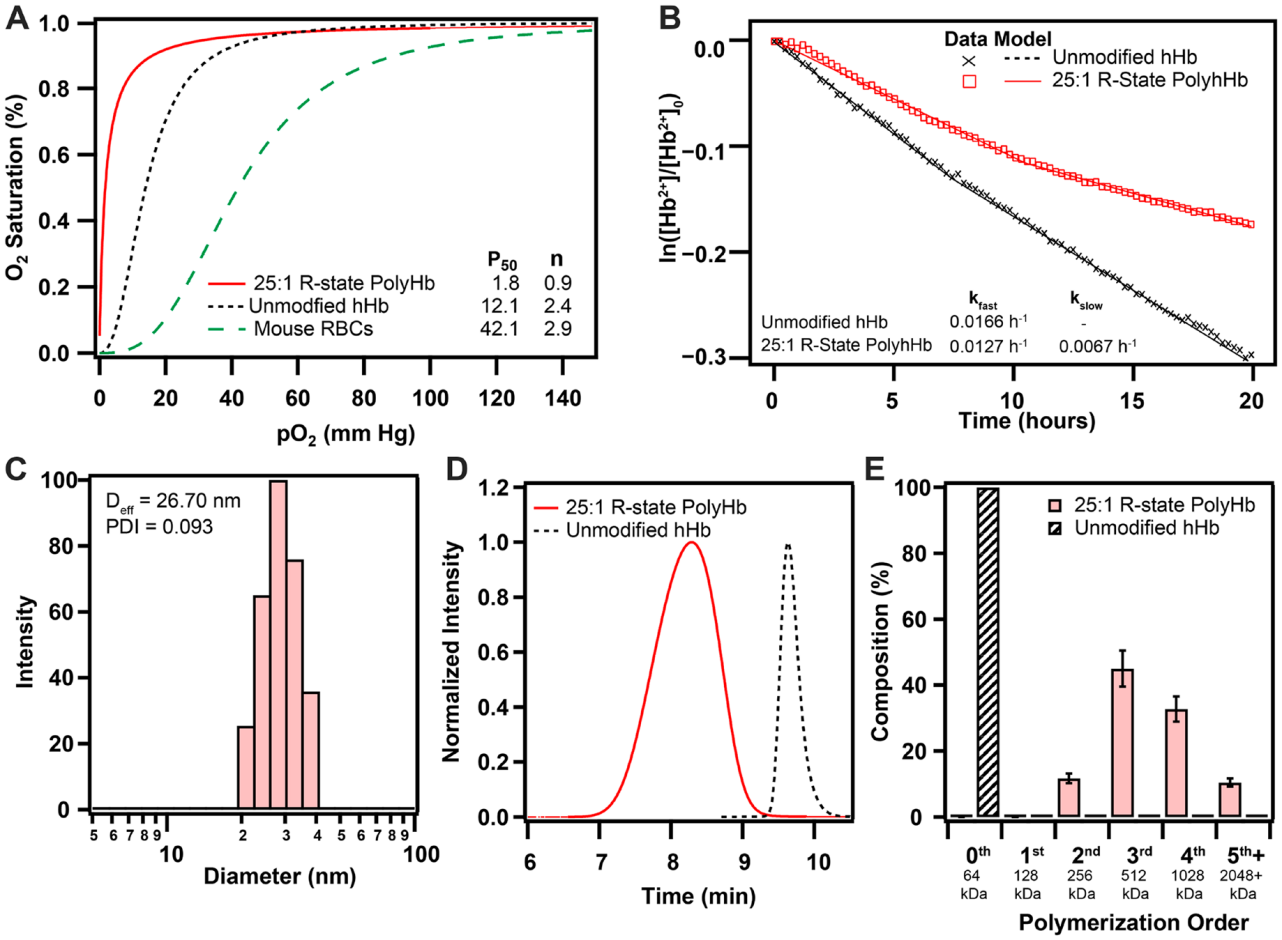
Biophysical properties of 25:1 R-state PolyHb used for this study. (**A**) O_2_ equilibrium curves for 25:1 R-state PolyHb, hHb, and mouse RBCs. (**B**) Auto-oxidation kinetics of hHb and 25:1 R-state PolyHb (1.25 g/dL). (**C**) Intensity distributions of hydrodynamic diameter of 25:1 R-State PolyHb. (**D**) Normalized SEC-HPLC intensity distributions of R-state PolyHb compared to unmodified hHb. (**E**) Polymer order distribution for 25:1 R-State PolyHbs. Polymer distribution was calculated on a percent by heme basis via analysis of the 413 nm absorbance wavelength. The corresponding approximate sizes of the polymer orders are shown below each group.

Polymerization of human hHb in the relaxed (R) quaternary state resulted in significant changes in its biophysical properties (Figure 1). Polymerization in the R-state significantly increased the O_2_ affinity (P_50_ = 1.8 ± 0.1 mm Hg) compared to hHb (12.1 ± 0.5 mm Hg). The O_2_ affinity of 25:1 R-state PolyHb was also much greater than mouse RBCs (42.1 ± 0.9 mm Hg). After polymerization, 25:1 R-state PolyHb no longer had a sigmoidal O_2_ binding curve, which indicates a loss of cooperative binding behavior. The effective hydrodynamic diameter of 25:1 R-state PolyHb was significantly larger (D_eff_ = 26.7 ± 3.2 nm) compared to unmodified hHb, as measured by Xu et al. (5 nm) [27]. Analysis of the chromatogram obtained with SEC-HPLC revealed the complete removal of extremely low MW PolyHb and unpolymerized Hb (128 kDa, 64 kDa) in the transfused sample. R-state PolyHb had significantly slower rates of auto-oxidation (k_fast_ = 0.0127 ± 0.0008 h^-1^, k_slow_ = 0.0067 ± 0.0003 h^-1^) compared to unmodified Hb (0.0166 ± 0.0009 h^-1^).

### ROS quantification

**Figure 2:**
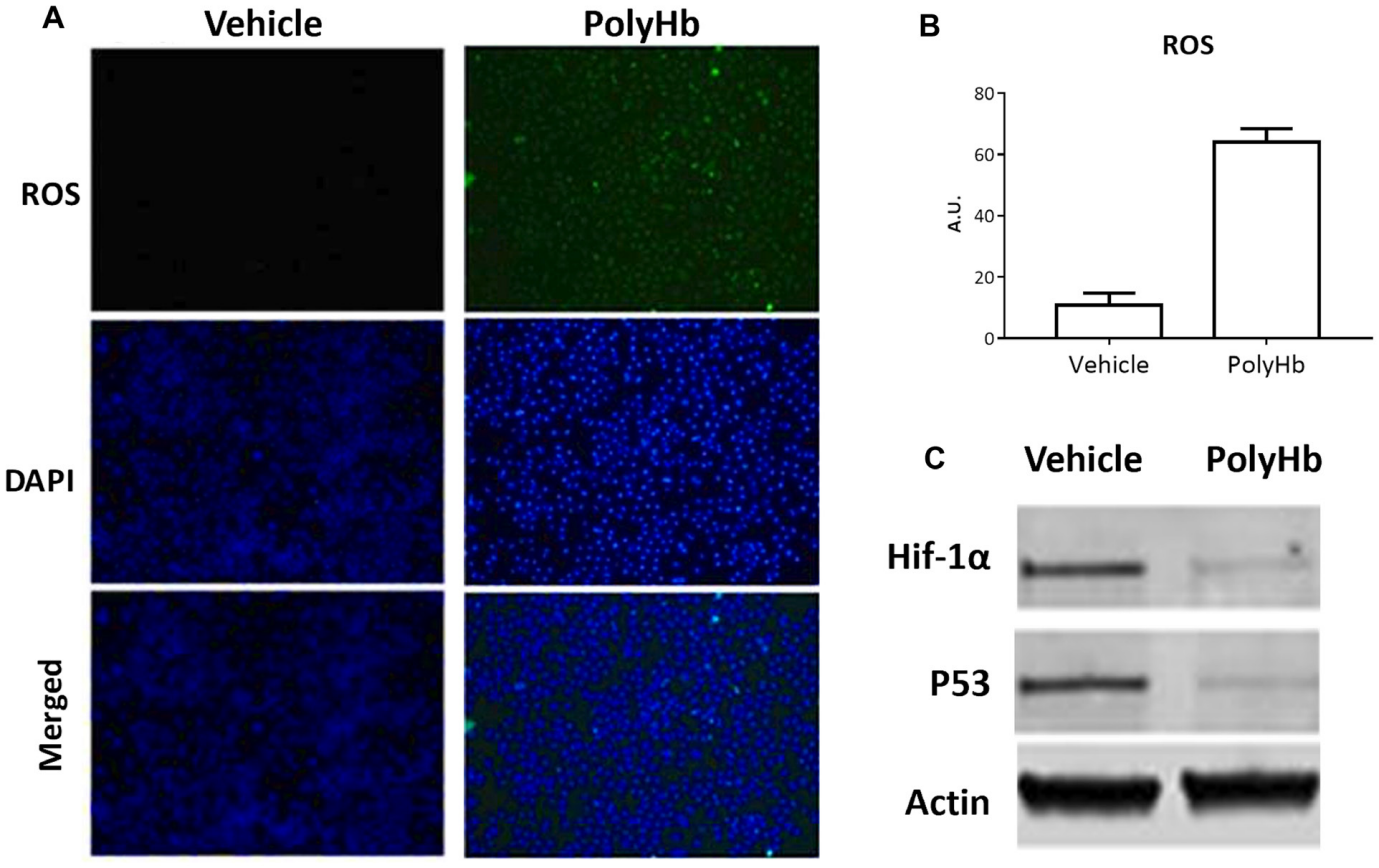
Effects of 0.05 g/dL PolyHb on ROS levels in A549 cells after 24 hours. (**A**) ROS (DCFH-DA) and DAPI stain are shown for PolyHb and a vehicle control. (**B**) ROS (DCFH-DA) fluorescent intensity of digested A549 cells after 24 hour exposure to PolyHb and a vehicle control. (**C**) Effects of PolyHb and a vehicle control on expression of HIF-1α, p53, and actin, as assessed by western blot.

Incubation of A549 cells with PolyHb for 24 hours resulted in a significantly increased expression of ROS relative to the control group (Figure 2A–2B). Incubation with PolyHb also reduced the expression of the hypoxia marker HIF-1α and the p53 oncogene (Figure 2C).

### Tumor cell viability in culture

**Figure 3:**
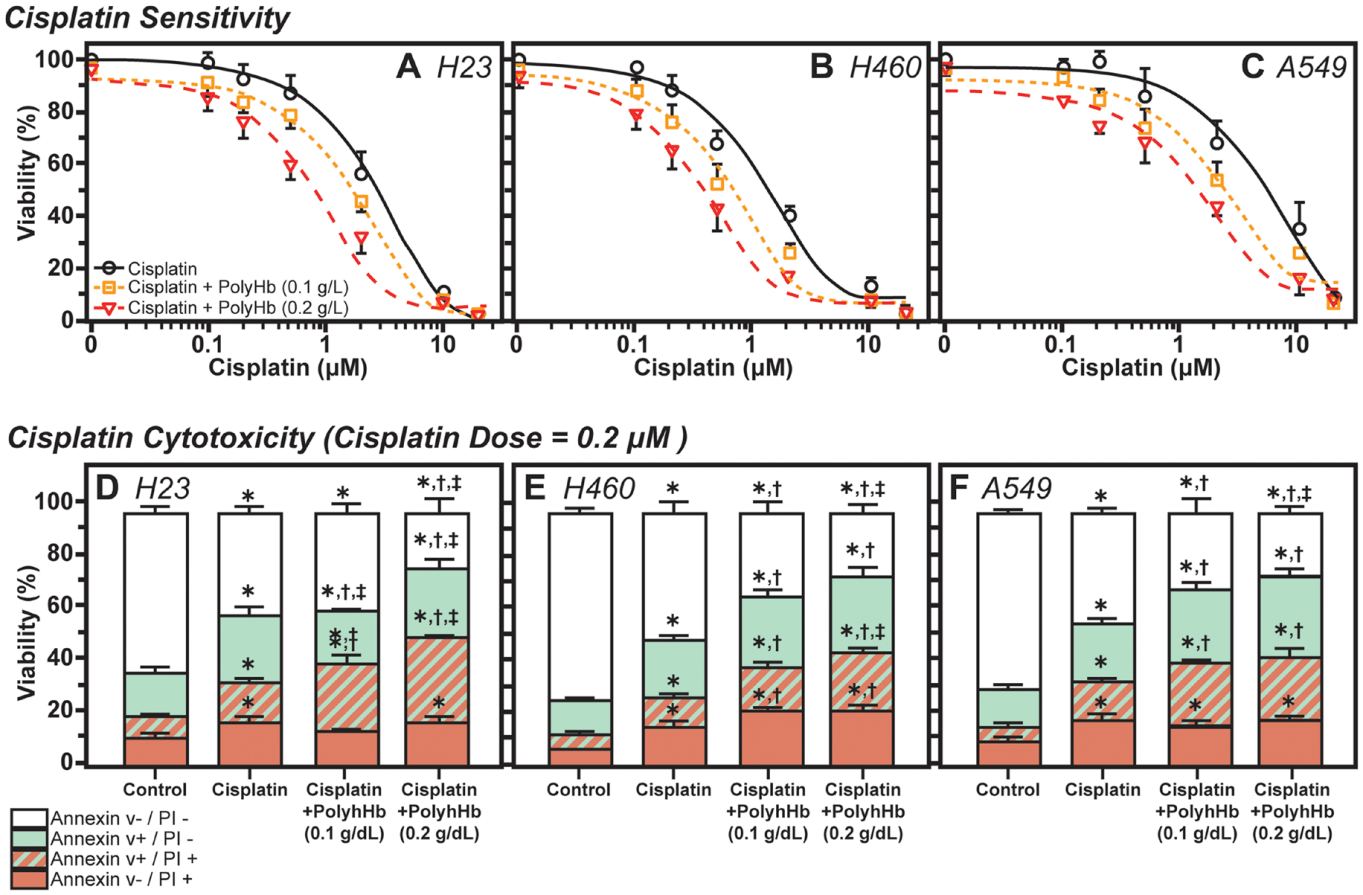
Effects of Cisplatin and PolyHb on cell viability and cytotoxicity. (TOP) Shift in cisplatin sensitivity in (**A**) H23, (**B**) H460, and (**C**) A549 cell lines upon addition of 0.1 or 0.2 g/dL of PolyHb. Curves represent the mean percentage of viable cells (mean ± SD) relative to 0 μM cisplatin (*n* = 5 total). (BOTTOM) Cisplatin cytotoxicity in (**D**) H23, (**E**) H460, and (**F**) A549 cell lines upon addition of 0.1 or 0.2 g/dL of PolyHb (mean ± SD, *n* = 5). The cisplatin dose was 0.2 μM in all groups treated for 48 h. ^*^significant with respect to the control group; ^†^significant with respect to the cisplatin-only group; ^‡^significant with respect to the cisplatin + PolyHb (0.1 g/dL) group.

The tumor cell viability in culture, as measured by the percentage of live cells after treatment with the three treatment groups, for the H24, H460, and A549 cell lines are shown in Figure 3. For H23 cells, the concentrations at which half of the cells became unviable (IC50) for cisplatin, cisplatin with PolyHb at 0.1g/dL, and cisplatin with PolyHb at 0.2 g/dL were 2.85 *μ*M, 1.69 *μ*M, and 0.80 *μ*M, respectively. For H460 cells, the IC50 for cisplatin, cisplatin with PolyHb at 0.1 g/dL and cisplatin with PolyHb at 0.2 g/dL were 1.31 *μ*M, 0.66 *μ*M, and 0.37 *μ*M, respectively. Finally, for H549 cells, the IC50 for cisplatin, cisplatin with PolyHb at 0.1 g/dL, and cisplatin with PolyHb at 0.2 g/dL were 5.27 *μ*M, 2.47 *μ*M, and 1.31 *μ*M, respectively. These results indicate that the largest decrease in cell viability during hypoxia, for the three studied cell types, was produced from treatment with cisplatin and PolyHb at a 0.2 g/dL concentration.

### Apoptosis and necrosis

The percentage of viable cells (negative for annexin V and PI), early apoptotic (positive for annexin V and negative for PI), late apoptotic (positive for annexin V and PI), and necrotic (positive for PI and negative for annexin V) cells after 48 h of treatment is presented in Figure 3. For H23 cells, treatment with both concentrations of PolyHb did not significantly increase the number of necrotic cells relative to the cisplatin-only treatment group. The presence of 0.2 g/dL PolyHb significantly increased the number of cells undergoing apoptosis relative to 0.1 g/dL PolyHb and control treatment groups. Relative to cisplatin-only treatment, the number of co-stained cells (undergoing apoptosis and were already necrotic) significantly increased in the presence of PolyHb. Furthermore, positive co-staining was proportional to the concentration of PolyHb. The 0.1 g/dL PolyHb group had significantly lower positive co-staining than the 0.2 g/dL group. Overall, the total number of viable cells, as determined by the negatively stained cells, was the same for both cisplatin alone and cisplatin with 0.1 g/dL PolyHb. The number of viable cells was significantly lower for cisplatin with 0.2 g/dL PolyHb.

For H460 cells, the presence of PolyHb, at both concentrations, significantly increased the number of both apoptotic and necrotic cells. While PolyHb concentration does not have an effect on the number of apoptotic cells or necrotic cells separately, the number of co-stained cells in the presence of 0.2 g/dL PolyHb was significantly higher than the number of co-stained cells in the presence of 0.1 g/dL PolyHb. Cisplatin combined with PolyHb had a significantly higher number of co-stained cells compared to cells treated with cisplatin alone. The number of viable cells followed an inverse trend to the positively co-stained cells. The most significant quantity of viable cells was present in the cisplatin-only group. A significantly lower number of viable cells were found when cisplatin was combined with 0.1 g/dL PolyHb. Finally, the least number of viable cells was found in the cisplatin combined with 0.2 g/dL PolyHb.

For A549 cells, a similar behavior to the H23 cells was observed. In the A549 cell line, the presence of PolyHb does not change the number of cells positively stained for necrosis, relative to cisplatin-only treatment. However, the presence of PolyHb significantly increased the number of cells undergoing apoptosis, relative to the cisplatin-only treatment. This increase was independent of PolyHb concentration. While there was a significant increase in the number of positively co-stained cells in the presence of PolyHb, there were no statistical differences between the two PolyHb concentrations. As with the other cell types, the highest number of viable cells was found in the cisplatin-only treatment group. A smaller number of viable cells was observed when cisplatin was combined with 0.1 g/dL PolyHb. The least number of viable cells was observed in the cisplatin combined with 0.1 g/dL PolyHb. All differences for the viable cells were statistically significant.

### *In vivo* tumor size

**Figure 4:**
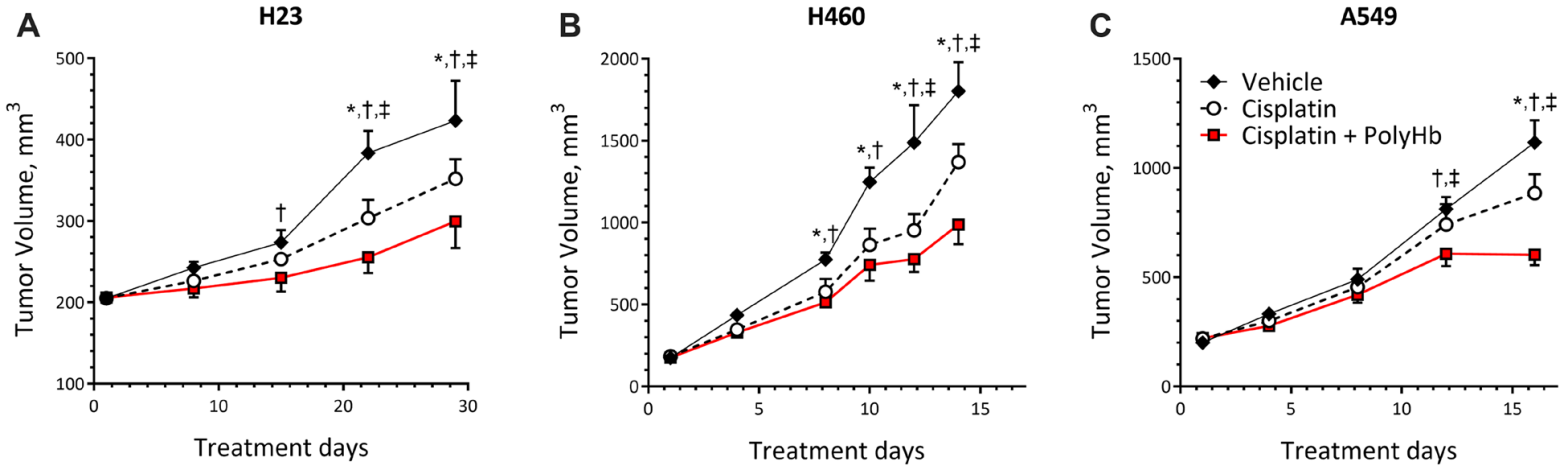
Tumor growth curves of (**A**) H23, (**B**) H460, and (**C**) A549 tumor grafts. Tumors were transplanted subcutaneously in the right flank of female BALB/c nude mice (*n* = 5). Tumor volume was calculated as (L × W)/2. Data represent the mean ± SD. ^*^ *p* < 0.05 compared to the control group; ^†^ *p* < 0.05 compared to the cisplatin-only group; ^‡^ *p* < 0.05 compared to the cisplatin + PolyHb (0.1 g/dL) group.

The changes in tumor size for mice implanted with H23, H460, and A549 cells followed by treatment with cisplatin-only, cisplatin-PolyHb combination, and vehicle control are shown in Figure 4. Regardless of treatment, each tumor cell line had different growth rates. H460 had the highest rate of growth, followed by A549 and then H23.

Starting at day 15 of treatment, H23 tumor volumes were significantly smaller when treated with cisplatin combined with PolyHb, compared to the vehicle control. This statistically significant smaller volume was preserved at days 22 and 29 of treatment. At these time points, the cisplatin-PolyHb combined treatment had significantly smaller tumor volumes compared to tumor volumes when treated with cisplatin alone. At days 22 and 29 of treatment, the cisplatin-only treatment group had statistically smaller tumor volumes compared to the vehicle group.

At days 8 and 10, H460 tumors treated with cisplatin-only and the cisplatin-PolyHb combined treatment group were significantly smaller compared to treatment with the vehicle control. At days 12 and 14 of treatment, the cisplatin with PolyHb treatment group had a significantly smaller tumor volume than the other two treatment groups, and the cisplatin-only group had a tumor volume only significantly smaller than that of the vehicle group.

The different treatments had no significant effect on A549 tumor volume until day 12 of treatment. On day 12, the vehicle and cisplatin-only groups had similar tumor volumes. The cisplatin with PolyHb treatment group had significantly smaller tumor volume compared to the other treatments. On day 16, the cisplatin with PolyHb group had significantly smaller tumor volume compared to the cisplatin-only and vehicle groups. At this time point, the cisplatin-only group had significantly smaller tumor volume compared to the vehicle group.

### *In vivo* HIF expression

**Figure 5:**
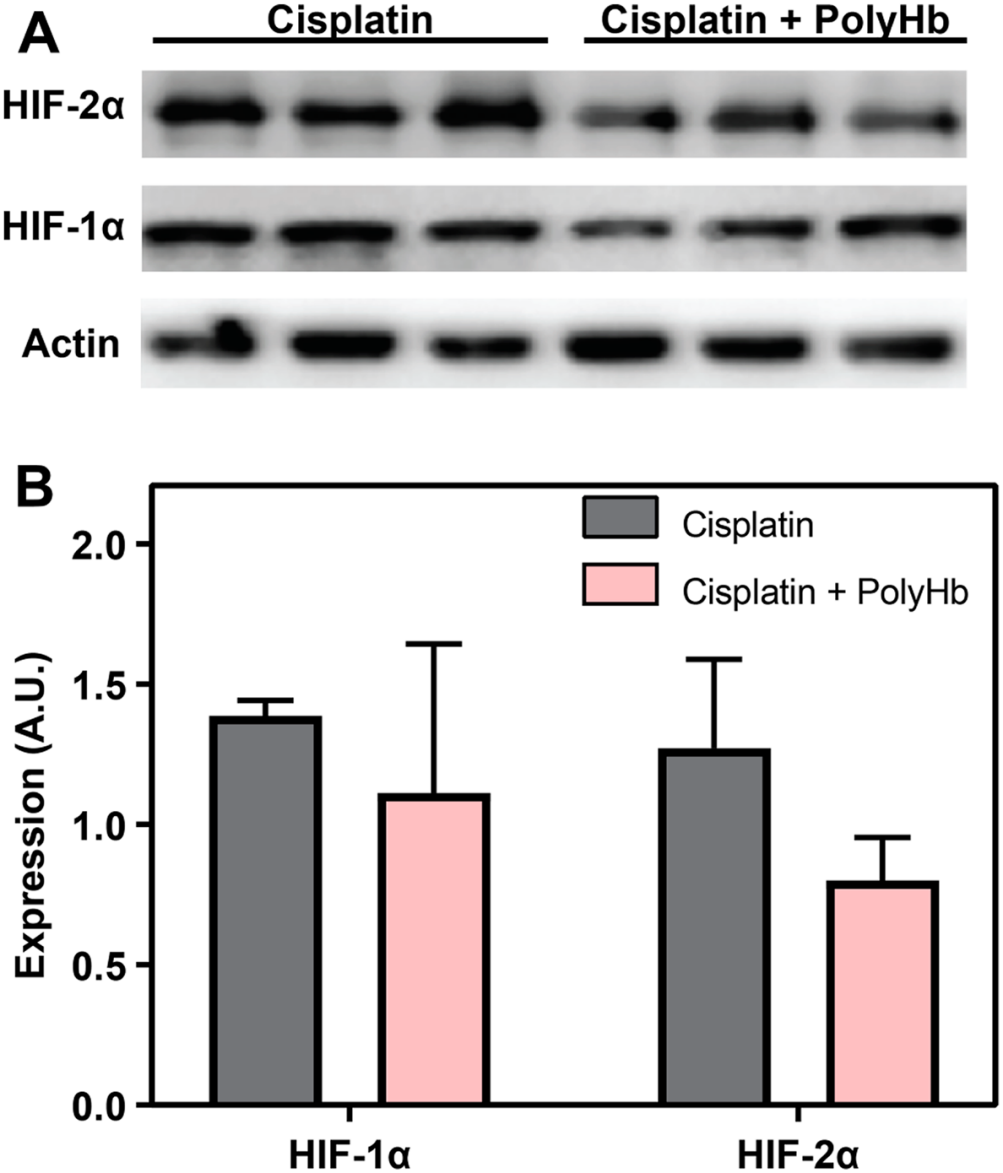
(**A**) Expression of HIF-1α and HIF-2α measured by Western blot analysis in the *in-vivo* A549 tumor. (**B**) Average intensity of the HIF-1α and HIF-2α bands for each experimental group, normalized to the vehicle control.

The expression of HIF-1α and HIF-2α measured by Western blot analysis in the A549 tumor is shown in Figure 5. Figure 5B shows the average intensity of the HIF-1α and HIF-2α bands for each experimental group, normalized to the vehicle control. While none of the differences were statistically significant, for both HIF-1α and HIF-2α, the presence of PolyHb caused a decrease in the amount HIF-1α and HIF-2α expression with PolyHb + cisplatin compared to the cisplatin only group.

## DISCUSSION

The principal finding of this study is that a high O_2_-affinity R-state PolyHb increases the sensitivity of NSCLC to cisplatin by promoting the formation of ROS and suppressing hypoxia-induced cisplatin resistance. These results are consistent with previous studies of HBOC facilitated chemosensitization [20–23]. Many previous studies attribute the sensitization of cisplatin to increased O_2_ delivery from the HBOC to the tumor [28–30]. While this hypothesis is part of the explanation, it does not fully explain our observations. The increased ROS expression after exposure to PolyHb in the A549 cells grown *in vitro* occurs despite the decrease in hypoxia markers HIF-1α and p53. This increase in ROS expression colocalized with dead cells (positively stained DAPI cells). Although the hypoxia markers were not overexpressed, ROS expression was still high for the PolyHb group. Therefore, the ROS may not be endogenously generated by the cells due to the hypoxic environment. Instead, these ROS may be generated by the PolyHb.

Weekly treatment with low MW PolyHb at low doses insufficient to increase blood O_2_ carrying capacity with short circulatory half-life (10 hours intravascular half-life) limits the oxygenation potential of PolyHb as a cisplatin sensitizing mechanism in NSCLC. In our previous study, we dosed every two days with higher concentrations of large MW PolyHb (24 hours intravascular half-life) to significantly increase oxygenation in breast tumors and directly induce O_2_ dependent antitumor activity [26, 31].

Free Hb can undergo auto-oxidation to methemoglobin (metHb), thereby producing free radicals [32]. Hb readily undergoes one-electron oxidations and reductions acting as a source and sink of free radicals. The auto-oxidation of heme produces O_2_^–^ and, indirectly, H_2_O_2_. Hb can react with redox-active xenobiotics and metabolites, forming xenobiotic and metabolite radicals that initiate a series of ROS generating reactions [33]. The oxidation of oxyhemoglobin produces O_2_^–^, metHb (HbFe^3+^), and ferrylhemoglobin (HbFe^4+^). MetHb loses heme at rates substantially higher than ferrylhemoglobin [34] and is converted to hemichrome forming Heinz bodies [33]. MetHb and ferrylhemoglobin reactions with radicals produce ferryl radicals with potent toxic effects [33]. This naturally occurring process inside red blood cells (RBCs) is suppressed by the highly specialized antioxidant system found in RBCs, which rapidly neutralizes ROS. Since many HBOCs are modified or cross-linked versions of acellular/cell-free Hb, the ROS generated from these auto-oxidation events cannot be readily neutralized through the endogenous RBC machinery [35]. High concentrations of low MW PolyHb, when used at high doses, induce coronary microcirculation toxicity due to oxidative stress [36]. Newer generations of HBOCs are coupled with antioxidants to minimize free radical formation [37]. The dramatic decrease in auto-oxidation of PolyHb and the precursor Hb solution compared to pure Hb likely results from copolymerization of superoxide dismutase and catalase that is present in the precursor Hb solution [38, 39].

The ROS generating ability combined with hypoxic reduction have a synergistic interaction. HIF-1α and p53 are fundamental mechanisms of cancer cells that reduce oxidative damage and prevent cell death [6]. Guo et al. showed that the hypoxia-induced cisplatin resistance in A549 cells is regulated by the expression of HIF-1α and p53, which actively promotes the molecular mechanisms that prevent the accumulation of damaging ROS inside cancer cells [6]. The PolyHb used in this study was in a N-acetyl-L-cysteine (NALC) modified Ringer’s Lactate solution. NALC is capable of slowly reducing hydrogen peroxide generated by the PolyHb species, which decreases oxidation of ferrous R-state PolyHb into the Fe^3+^ state [40]. However, it is insufficient to fully attenuate ROS generation that may damage cells at the cell interface.

Therefore, PolyHb increases the activity of cisplatin by preventing the expression of HIF-1α and p53 through moderately increasing the level of O_2_ available to the cell, while exogenously contributing to ROS levels. This hypothesis has been proposed and validated by previous research which demonstrated that increased ROS production reduces the hypoxia-induced cisplatin resistance *in vitro* [7]. Hb mediated iron and ROS can be considered as potential anti-cancer agents alone or when combined with chemotherapy, as both alter cancer cell mitochondrial oxidative metabolism resulting in increased levels of O_2_^-^ and H_2_O_2_ [41]. The increased *in vitro* cisplatin sensitivity in the presence of PolyHb was apparent in the sensitivity curves shown in Figure 3. While an in-depth ROS analysis, such as the one shown in the A549 cells, was not carried out for H23 and H460 cells, the increased *in vitro* sensitivity to cisplatin in these two additional cancer cell types can be attributed to similar mechanisms. Furthermore, these sensitivity curves also show that the increase in sensitivity is dependent on the concentration of PolyHb. The PolyHb concentration-dependent effect of cisplatin sensitization is likely due to increased ROS formation in the presence of PolyHb. The increase in PolyHb concentration may also increase O_2_ availability to the cells. However, because HIF-1α and p53 expression in A549 cells was already decreased after exposure to 0.05 g/dL PolyHb, further increases in the PolyHb concentration may not lead to significant hypoxia reduction. This suggests that differences in sensitivity between the two PolyHb concentrations result primarily from the increased formation of ROS at higher PolyHb concentrations.

Apoptotic and necrotic markers are also affected by PolyHb addition to cisplatin-treated cells. The addition of cisplatin alone significantly increased the number of cells that are apoptotic, necrotic, or a combination of the two. This increase in apoptosis is consistent with the known increase in cancer cell apoptosis in the presence of cisplatin [42]. Cisplatin’s mechanism of action involves DNA cross-linking and damage, which results in the initiation of cellular apoptotic pathways [14]. Other studies suggest that cisplatin’s cytotoxicity involves the formation of mitochondrial ROS, which leads to the initiation of apoptotic pathways through mitochondrial membrane damage and widespread cellular damage [15–19]. In hypoxia, upregulation of HIF-1α and p53 promotes the reduction of ROS, leading to cisplatin resistance [6, 7]. Therefore, PolyHb’s ability to prevent HIF-1α and p53 expression *in vitro*, as shown in the A549 cells, increases the availability of ROS capable of contributing to apoptotic pathways.

Furthermore, PolyHb is capable of contributing exogenous ROS to the cells, which can further promote apoptotic mechanisms. This may be the mechanism for the increased apoptosis observed in the presence of cisplatin and PolyHb, compared to cisplatin alone. For H460 and the A549 cells, there was no significant increase in apoptosis when doubling the PolyHb concentration. However, the number of viable cells was significantly lower when exposed to a higher PolyHb concentration. The decrease in viable cells suggests that moderate increases in apoptosis and necrosis were promoted by the increased PolyHb concentration. Thus, the increase in PolyHb derived ROS is likely the cause of increased cell death relative to increased O_2_ delivery. Further analysis of the apoptotic and necrotic pathways is required to confirm any solid conclusion.

*In vitro* and *in vivo* sensitization of NSCLC cisplatin sensitization via PolyHb were similar. PolyHb and cisplatin synergistically significantly decreased tumor volume. At the end of the measurement period for each tumor type, the tumor volume after transfusing cisplatin-PolyHb together was always significantly less than the volume of the cisplatin-only and vehicle groups. Semiqualitative Western blot analysis of A549 implanted tissue demonstrated that, while not statistically significant, there was a decrease in the expression of HIF-1 and HIF-2 in the presence of PolyHb. This suggests that PolyHb had a minimal effect on reducing the hypoxic environment of the tumor. However, it is unlikely that this was the primary mechanism of cisplatin sensitization *in vivo*.

Other small MW HBOCs, such as YQ23, leave the intravascular compartment into the tumor microenvironment, which directly increases O_2_ availability to the tumor [22]. The extravasation of YQ23 has been attributed to its ability to decrease tumor volume [22]. Measurements of tumor pO_2_ with YQ23 show a significant increase in tumor O_2_ tension (up to 5 mm Hg) relative to the absence of the carrier at doses of 0.2 g/kg and 0.4 g/kg [20, 22]. In these studies, YQ23 intravascular peak concentrations were between 0.3 g/dL and 0.8 g/dL assuming normal mice body weights [20, 22]. Although YQ23 is a different configuration of tetrameric Hb, the O_2_ carrying capacity is the same as tetrameric Hb (1.36 mLO_2_/gHb). Given the low solubility of O_2_ in blood (0.0031 mLO_2_/mm Hg/dL at 37°C) in plasma, the maximum resulting increase in pO_2_ for the peak intravascular concentrations of YQ23 should be 2.7 mm Hg/dL and 5.4 mm Hg/dL, respectively. For the two studies 0.43 mm Hg/min and 0.22 mm Hg/min of oxygen would be delivered to the tissues respectively, given an approximate cardiac output in mice equivalent to 8 mL/min. Using an endogenous Hb concentration of 15 g/dL, the baseline O_2_ delivery in mice is about 11 mm Hg/min. From this analysis, the increase in O_2_ delivery provided by YQ23 is minimal, and unlikely to cause the increases in tumor O_2_ reported [20, 22]. Meeting the increase in O_2_ delivery would require that YQ23 to be exclusively releasing O_2_ to the tumor, for more than 10 minutes, without any O_2_ consumption by other tissues. If a similar analysis is carried out with the PolyHb used in this study with a dose of 1.6 g/kg (3.75 × dose used in [22]), then the additional O_2_ delivery supported by PolyHb would be 1.6 mm Hg/min. This amount is only ~14% of the endogenous O_2_ delivery of the animal. While both YQ23 and PolyHb may have physical properties that might allow for localization of the molecules in the peritumor area it is an oversimplification to assume that HBOCs will exclusively release O_2_ to the peritumor area if the HBOCs accumulate in that location.

Given that HBOCs accumulate in the peritumor area, Hb auto-oxidation is likely to cause the most significant increase in ROS in these regions. Hb, under physiological conditions (i.e., inside the RBC), autoxidizes at a rate of about 3% per day [43]. Outside the RBC, with the influence of external factors such as varying pH and ionic concentrations, this oxidation rate is greatly affected. As presented in [44], the rate of auto-oxidation of human Hb is faster at more acidic pHs. The auto-oxidation of HBOCs is likely to increase in the tumor microenvironment due to increased acidity resulting from increased anaerobic glycolysis and the presence of proton pumps that promote an acidic environment [45]. Therefore, the accumulation of HBOCs in the tumor microenvironment and a likely increase in Hb auto-oxidation indicate that increased ROS generation and accumulation can lead to increased damage to the cancer cells in the tumor, thus explaining the decreased tumor volumes in the presence of PolyHb and cisplatin.

This study serves as preliminary evidence of PolyHb’s ability to increase the sensitivity to cisplatin in NSCLC. However, these results are limited because the exact mechanism of sensitization is still unclear. The evidence presented in this study suggests that decreased hypoxia due to facilitated O_2_ delivery combined with endogenous O_2_ delivery in the animal might have a small effect on cisplatin sensitization. However, a second, but more likely mechanism, auto-oxidation of PolyHb in the tumor microenvironment can lead to the formation of ROS that can enhance the cytotoxicity of cisplatin. Future studies should aim at utilizing *in vivo* approaches for both ROS detection [46] and intravascular oxygenation [47] in the implanted tumors, as an approach for determining the effects of PolyHb in these two essential processes. Additionally, these studies should consider incorporating superoxide disumatase and catalase to evaluate the exact modes of ROS generation that influence the chemosensitization mechanism as a function of environmental O_2_ tension. Future studies should also explore how administration of PolyHb solutions modulate proly hydroxylase (PHD), gene expression, and protein expression after extended doses.

## MATERIALS AND METHODS

### Polymerized hemoglobin preparation and analysis

R-state PolyHb was prepared from human Hb (hHb) extracted from human RBCs as described previously [48]. After purifying the material, we measured the biophysical properties of the PolyHb [31]. The cyanomethemoglobin method was used to measure the Hb concentration and the methemoglobin (metHb) level of hHb/PolyHb solutions [49, 50]. The size distribution by volume of PolyHb was measured using dynamic light scattering (DLS) (Brookhaven Instrument Inc. BS-200M, Holtsville, NY). The O_2_-hHb/PolyHb equilibrium binding curves were generated using a Hemox Analyzer (TCS Scientific Corp., New Hope, PA). The rheology of PolyHb plasma mixtures was measured using a DV3T-CP cone and plate rheometer (Brookfield AMETEK, Middleboro, MA) with cone spindle CPA-40Z [26]. The MW distribution was estimated with an Acclaim SEC-1000 column (Thermo Scientific, Waltham, MA) connected to a Thermo Scientific Dionex Ultimate HPLC/UHPLC system [38]. The flow rate of the mobile phase (0.5 mM 7.4 phosphate buffer) was maintained at 0.35 mL/min for all runs. Full-spectrum UV-visible (200 to 700 nm) absorbance spectra were collected for the duration of each run. All quantitative measurements of PolyHb composition were performed from the absorbance at 413 nm. To estimate the MW distribution of low MW PolyHb, we used the manufacturer provided calibration of the SEC column corrected for the holdup time in the tubing between system components. Auto-oxidation of ferrous PolyHb (Fe^2+^) (1.25 g/dL) to ferric PolyHb (Fe^3+^) was monitored at 37°C over 24 hours in a temperature controlled HP 8452 photodiode array spectrophotometer. The resulting spectra were assessed with multicomponent analysis and a two phase linear fit [38] using the segmented fit package in R 3.6.1.

### Cell culture and viability

NSCLC cell lines A549, H23, and H460 were obtained from ATCC (American Type Culture Collection, Manassas, VA, USA). Cells were grown as monolayers and maintained in the exponential growth phase at 5% CO_2_ in a humidified incubator at 37°C under normoxic conditions. Cells were cultured in DMEM (10% FBS, 100 U/mL penicillin, 100 μg/mL streptomycin, and 2 mM L-glutamine) (Life Technologies, Carlsbad, CA, USA). Cisplatin [cis-DDP, cis-diamminedichloroplatinum (II), P4394] was purchased from Sigma Aldrich (St Louis, MO, USA). A stock solution of 5 mmol/L cisplatin was prepared in 0.9% NaCl and stored at −80°C. Dilutions were made in PBS. Hypoxic conditions (5% O_2_/5% CO_2_) were achieved by connecting a ProOx O_2_ controller (BioSpherix, Redfield, NY) to the incubator. For *in vitro* treatments, cells were initially seeded in 96-well plates and after overnight incubation under hypoxic conditions, PolyHb at a final concentration of 0.05 g/dL was added in the culture medium and cells were cultured for an additional 24 h. Similarly, for the *in vitro* cytotoxicity test, cells were pre-seeded into 96-well plates (1 × 10^5^ per well) and incubated overnight under hypoxic conditions. Then, cells were cultured for an additional 48 h with and without PolyHb at concentrations of 0.1 and 0.2 g/dL in the culture medium, and with different concentrations of cisplatin. A standard methyl thiazolyltetrazolium (MTT, Sigma-Aldrich) assay was used to determine relative cell viability. Six replicates were completed for each of the experimental groups (*n* = 6).

### Reactive oxygen species

Dichlorofluorescindiacetate (DCFH-DA) was used to qualitatively detect the formation of ROS. Non-fluorescent DCFH-DA is oxidized to fluorescent dichlorofluorescein (DCF) by ROS. Cells were plated at 1 × 10^5^ in a confocal dish and incubated for 24 h. Cells were treated with vehicle (0.9% saline) and PolyHb and further incubated with DCFH-DA (10 μM) for 15 min before imaging. Glass dishes were observed by confocal laser scanning microscopy (CLSM). Quantitative analysis of intracellular ROS was conducted after cells were digested and collected by centrifugation (1000 rpm, 5 min), and quickly the fluorescence intensity was measured using a Spectra Max M5 spectrofluorometer (Molecular Devices, Sunnyvale, CA, USA) at an excitation of 485 nm and emission of 535 nm.

### Annexin V/propidium iodide staining

Cells were plated at 5 × 10^4^ cells/well in 24-well plates and allowed to attach overnight. PolyHb at concentrations of 0.1 and 0.2 g/dL with and without cisplatin at 0.2 μM were added to the culture medium. After 48 hours, cells were harvested by trypsinization, centrifuged, and resuspended in 50 μL of Annexin V in binding buffer (10 mmol/L N -2-hydroxyethylpiperazine-N -2-ethanesulfonic acid, pH 7.4; 150 mmol/L sodium chloride, 5 mmol/L potassium chloride; 1 mmol/L magnesium chloride; and 1.8 mmol/L calcium chloride). After 10 minutes of incubation at 4°C, 150 μL of annexin V binding buffer and 20 μL of propidium iodide (100 μg/mL in PBS) were added, and flow cytometry (FACScan; BD Immunocytometry Systems, San Jose, Calif) was performed. For every experiment, a minimum of 1 × 10^4^ cells was analyzed, and single- and double-labeled cells were counted, and the percentages of cells labeled with annexin V and/or propidium iodide were calculated. All experiments were performed with 6 replicates (*n* = 6).

### Western blot analysis

Cells were treated with either vehicle (0.9% saline) or PolyHb for 24 h under hypoxic conditions. Total cell extracts were prepared by incubating cells at 4°C for 30 min in lysis buffer and a mixture of protease inhibitors (Sigma-Adrich). Proteins were transferred to polyvinylidene difluoride (Merck Millipore, Burlington, MA) or nitrocellulose (Biorad) membranes. The following antibodies were used: P53 (Cell Signalling Technology, Danvers, MA, no. 9282; 1:1000); and HIF-1α (Abcam, 1:1000, ab82832), HIF-2α (Santa Cruz, sc-28706); and Actin (Sigma-Aldrich). Image J software was used to quantify the relative density of the Western blot bands and was presented as an adjusted relative density (area sample/area standard/area loading control sample/area loading control standard).

### Animal preparation

All protocols were approved by the Institutional Animal Care and Use Committee of the University of California, San Diego, and conducted according to the Guide for the Care and Use of Laboratory Animals (US National Research Council, 2011). All cells were tested for mycoplasma prior to inoculation in mice. Female BALB/c nude mice (22 ± 3 g) were subcutaneously injected with 5 × 10^6^ of A549 cells suspended in PBS and containing 50% Matrigel Matrix (Coining, 354234). Female athymic nude (Foxn1^nu^) mice (20 ± 2 g) were subcutaneously injected with 1 × 10^7^ H23 cells or 5 × 10^6^ H460 cells into the right hind flank suspended in PBS and Matrigel Matrix (Coining, 354234) to establish additional NSCLC xenograft models. Mice began receiving treatment once tumors reached approximately 200 mm^3^ (± 15%). Tumor volume was calculated as length × width × width/2. Cisplatin was administered intraperitoneally twice a week (q7d × 2, IP) at 3 mg/kg (at 8 mL/kg) and PolyHb was administered IV through the tail vein once a week.

### Statistical analysis

Values are presented as means and standard deviations (SD). The data were analyzed and plotted using Graphpad. Student’s *t*-test (two-tailed) or one-way ANOVA was performed to determine significance when comparing data from different treatment groups. *P* values were calculated, and *P* < 0.05 was considered to represent a significant difference.

## CONCLUSIONS

This study confirms that HBOCs combined with an anticancer drug can enhance the sensitivity of the tumor to chemotherapy, suggesting a new approach for treating patients suffering from NSCLC tumors. Therefore, infusing PolyHb as a co therapeutic may lead to reduced chemotherapeutic doses, which can reduce chemotherapeutic toxicity while improving the efficacy of the current standard-of-care. Hence, we believe that PolyHb combined with an anticancer drug is a promising approach towards tumor oxygenation and ROS formation, therefore increasing the sensitivity of chemotherapy in tumor treatment. The resulting improvement in therapeutic outcomes will be useful and practical in clinical situations.
